# Biomechanical and clinical analysis of pelvic stability and the role of internal fixation after debridement for sacroiliac joint tuberculosis with different degrees of bone destruction

**DOI:** 10.3389/fbioe.2026.1808745

**Published:** 2026-07-21

**Authors:** Long Ma, Haiyang Cheng, Qiang Liu, Kun Wang, Zhangui Gu, Jiandang Shi, Jianfeng Xu, Zongqiang Yang

**Affiliations:** 1 Department of Orthopedic, General Hospital of Ningxia Medical University, Yinchuan, China; 2 First Clinical Medical College of Ningxia Medical University, Yinchuan, China

**Keywords:** sacroiliac joint tuberculosis, pelvic stability, finite element analysis, lesion debridement, internal fixation, bone destruction

## Abstract

**Objective:**

To investigate the biomechanical characteristics and clinical outcomes after lesion debridement at varying degrees of sacroiliac joint destruction, and to assess the role of internal fixation in postoperative stability.

**Materials and Methods:**

Finite element analysis: An intact three-dimensional finite element model of the pelvis was established based on CT data from a healthy adult. On this basis, unilateral sacroiliac joint articular surface destruction of 25%, 50%, 75%, and 100% was simulated. For each destruction level, two postoperative models were constructed: debridement alone and debridement combined with internal fixation. Under physiological standing load conditions, pelvic global displacement, relative displacement of the sacroiliac joint surface, and displacement and stress distributions of the sacrum and ilium were comparatively analyzed among different models. Clinical analysis: A retrospective analysis was performed on 20 patients with sacroiliac joint tuberculosis who underwent lesion debridement at our institution between January 2010 and December 2025. Based on preoperative imaging, all patients had mild sacroiliac joint destruction (<1/3 involvement). The patients were divided into a debridement-alone group (n = 10) and debridement combined with internal fixation group (n = 10). Perioperative variables, including operative time, blood loss, and inflammatory markers, were systematically compared between the two groups. Clinical efficacy was evaluated by dynamically assessing changes in visual analogue scale (VAS) pain scores and Majeed functional scores.

**Results:**

Finite element analysis demonstrated that, in the debridement-alone model, overall pelvic displacement, relative displacement across the sacroiliac joint, as well as local bone displacement and stress values increased progressively with the severity of sacroiliac joint destruction. Under mild destruction, changes in these biomechanical parameters were minimal and showed no significant differences compared with the intact model (*P* > 0.05). However, under moderate-to-severe destruction, all parameters increased significantly (*P* < 0.05), indicating a marked reduction in both global and local pelvic stability. Following the addition of internal fixation, overall pelvic displacement, local stress, and relative joint displacement were all significantly reduced under moderate-to-severe destruction conditions. Clinical results showed that, in patients with mild bone destruction, there were no significant differences between the debridement-alone group and the debridement combined with internal fixation group in terms of pain relief, functional recovery, or radiographic improvement during follow-up (*P* > 0.05). Both groups achieved satisfactory clinical outcomes, with no evident recurrence or complications.

**Conclusion:**

This study provides an initial biomechanical arithmetic modeling framework and compares its outputs with relevant clinical findings. The degree of sacroiliac joint destruction is closely associated with postoperative pelvic stability. In cases of mild destruction, debridement alone yields satisfactory outcomes, with no additional benefit observed from adjunctive internal fixation. As the severity of destruction increases, pelvic stability progressively declines, and the stabilizing effect of internal fixation becomes increasingly evident.

## 1 Introduction

Sacroiliac joint tuberculosis (SJT) is a relatively uncommon form of osteoarticular tuberculosis, predominantly presenting with unilateral involvement (approximately 90%), while bilateral involvement is relatively rare (approximately 10%) (Prakash, 2014). However, due to its concealed anatomical location and the lack of specific clinical symptoms in the early stage, varying degrees of articular surface destruction and bone defects are often present at the time of diagnosis (Jain et al., 2022). With disease progression, the structural integrity of the sacroiliac joint is gradually compromised, which may lead to a reduction in pelvic stability and subsequently increase the risk of pain, functional impairment, and secondary deformities. For patients with pronounced bone destruction or poor response to conservative treatment, lesion debridement with reconstruction of the local structure is generally considered a necessary therapeutic approach (Zhu et al., 2017). However, residual bone defects after lesion debridement may alter the mechanical load transmission of the sacroiliac joint and the pelvic ring. Whether concomitant internal fixation is required to maintain postoperative stability, particularly in cases without complete joint destruction, remains controversial. In clinical practice, the application of internal fixation is often based on subjective assessment of the extent of destruction or the surgeon’s experience, and its biomechanical rationale has not yet been adequately quantified or validated.

The sacroiliac joint plays a critical role in axial load bearing and the maintenance of overall pelvic stability, and its stability primarily depends on the combined effects of articular surface interlocking, osseous support, and the surrounding ligamentous structures (Kondratiev et al., 2025). When the continuity of the articular surface is disrupted, local stress distribution and displacement patterns are altered, which may exert an amplified effect on the overall mechanical behavior of the pelvic ring. Previous finite element studies have investigated the biomechanical characteristics of the sacroiliac joint in degenerative diseases, traumatic conditions, and after fusion procedures (Lindsey et al., 2018; Enix and Mayer, 2019; Joukar et al., 2019). However, biomechanical studies focusing on progressive bone destruction caused by tuberculous lesions and postoperative residual defects remain limited.

Finite element analysis (FEA) allows the simulation of mechanical responses under different degrees of bone destruction and reconstruction strategies under controlled conditions, providing an effective tool for the quantitative evaluation of postoperative stability (Verma et al., 2024; Zhang Y. et al., 2024). Based on this, the present study constructed graded destruction models according to the extent of sacroiliac joint involvement and simulated two postoperative conditions, namely, lesion debridement alone and lesion debridement combined with internal fixation. Stress distribution and changes in stability under physiological loading conditions were systematically analyzed. The study aimed to clarify the effect of internal fixation on postoperative mechanical stability at different degrees of destruction and to identify a potential destruction threshold requiring internal fixation, thereby providing objective biomechanical evidence to guide surgical strategies for sacroiliac joint tuberculosis.

## 2 Materials and methods

### 2.1 Finite element analysis

#### 2.1.1 Intact pelvic finite element model

A healthy 30-year-old female volunteer (height: 168 cm; weight: 60 kg) with no history of pelvic disease, trauma, or surgical intervention was selected for this study. X-ray examination showed no evident degenerative changes in the sacroiliac joints. Computed tomography (CT) scanning of the pelvis and partial femora was performed using a 64-slice spiral CT scanner (Philips, Amsterdam, Netherlands) with a slice thickness of 1.5 mm. Image data of the intact pelvis and partial femora were obtained and exported in DICOM format. Three-dimensional reconstruction of the pelvis was completed in Mimics 21.0 (Materialise NV, Leuven, Belgium). Pelvic and partial femoral surface models were generated through image segmentation and calculation and exported as STL files. The STL files were then imported into 3-Matic 13.0 (Materialise NV, Leuven, Belgium) for geometric optimization, including model repair, noise removal, smoothing, surface optimization, and mesh processing, to improve model continuity and geometric accuracy and to meet the requirements of finite element analysis. Meanwhile, the internal fixation system (plates and screws) was established in 3-Matic and assembled onto the pelvic model according to the clinical surgical position. Finally, the intact pelvic model was exported in MXP format and imported into ANSYS (ANSYS, Inc., USA) for assignment of material properties, application of boundary conditions and loads, and analysis of displacement and stress distribution under different loading conditions.

**FIGURE 1:**
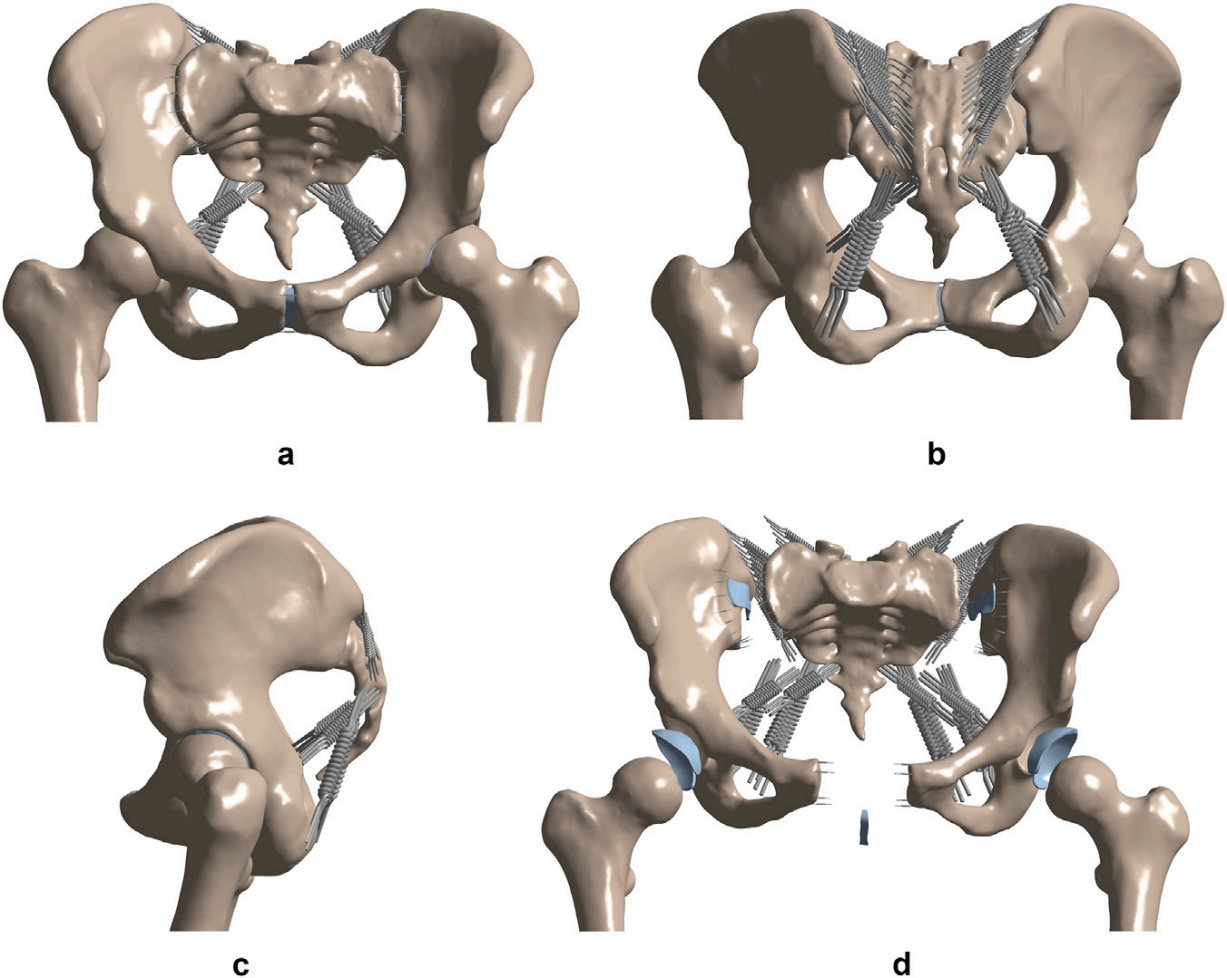
Anatomical structures of the pelvic finite element model. **(a–c)** Anterior, posterior, and lateral views of the pelvic finite element model. **(d)** Exploded view of the pelvic finite element model.

The intact pelvic finite element model is shown in Figure 1. The bony structures of the pelvis were modeled with separate cortical and cancellous bone layers, with the cortical bone thickness uniformly set to 1.0 mm. Articular cartilage was applied to the sacroiliac joint and acetabular regions, and the cartilage structures were established separately in the model. Figure 1d presents an exploded view of the intact pelvic model, clearly illustrating the sacrum, bilateral ilia, femora, as well as the spatial relationships between the sacroiliac joint cartilage and acetabular cartilage. Eight major ligaments contributing to pelvic stability were simulated using spring elements, with their stiffness values and quantities listed in Table 1 (Zhang J. et al., 2024; Liu et al., 2026). These ligaments included the sacrospinous ligament (SSL), sacrotuberous ligament (STL), interosseous sacroiliac ligament (ISL), anterior sacroiliac ligament (ASL), short posterior sacroiliac ligament (SPSL), long posterior sacroiliac ligament (LPSL), superior pubic ligament (SPL), and arcuate pubic ligament (APL). During mesh generation, both computational accuracy and efficiency were considered, and a global element size of 1.0 mm was applied. The intact pelvic finite element model consisted of a total of 1,363,634 elements.

**TABLE 1:** Ligament stiffness and quantities used in the pelvic finite element model.

Ligament	Stiffness K (N/mm)	Quantity
Sacrospinous ligament	1,400	5
Sacrotuberous ligament	1,500	5
Interosseous sacroiliac ligament	2,800	4
Anterior sacroiliac ligament	700	10
Long posterior sacroiliac ligament	1,000	4
Short posterior sacroiliac ligament	400	10
Superior pubic ligament	500	2
Arcuate pubic ligament	500	2

#### 2.1.2 Validation of the finite element model

To validate the pelvic finite element model, physiological loading conditions were applied to the model in ANSYS. The inferior surfaces of both femora were fixed, and a 500 N axial load was applied to the superior surface of the sacrum (S1) to simulate body weight during quiet standing. In addition, a torque of 7.5 Nm was applied at S1 to induce flexion, extension, lateral bending, and axial rotation, thereby simulating different physiological motion states. Model validation was performed by comparing the stress distribution and displacement characteristics of the intact pelvis, as well as the range of motion (ROM) of the sacroiliac joint, with results from previously published *in vitro* experiments or finite element studies (Lindsey et al., 2014; Hu et al., 2017; Cross et al., 2018; Zhang J. et al., 2024).

#### 2.1.3 Finite element postoperative model

Based on the intact pelvic finite element model, postoperative finite element models of the sacroiliac joint were further constructed. The degree of sacroiliac joint destruction was graded according to the involved area of the left sacroiliac cartilage as 25%, 50%, 75%, and 100%. At the same time, destruction of the adjacent local bone and related ligamentous structures surrounding the cartilage was considered to reflect the comprehensive damage characteristics of tuberculous lesions in the sacroiliac joint. On the basis of these destruction models, two primary postoperative models were established after simulating lesion debridement and the resulting postoperative structural condition: a lesion debridement-alone model and internal fixation model. Previous analytical studies on porous materials have shown that cavities or inclusions with different geometries may influence the mechanical response of materials (Panteliou and Dimarogonas, 1997a; 1997b). The debrided region was simplified as a mechanically non-load-bearing defect rather than a biologically empty cavity; clinically, it was filled with particulate bone grafts and may contain postoperative fluids, such as blood, tissue fluid, and residual irrigation fluid, but their immediate mechanical contribution was not considered because they provide limited immediate structural support under the current quasi-static loading conditions (Brink, 2021; Wang, 2023). The internal fixation model adopted a double-internal fixation configuration. The plates had a thickness of 3.5 mm, a width of 10 mm, and a length of 60 mm. All screws had a diameter of 3.5 mm, with lengths of 12 mm, 18 mm, 20 mm, and 30 mm, respectively. The plates and screws were modeled in 3-Matic and assembled onto the pelvic model according to commonly used clinical fixation positions to ensure the rationality of their anatomical placement and spatial relationships. Mechanically, the plate-screw construct was modeled as a bridging fixation structure across the debrided sacroiliac region. Its stabilizing effect depends not only on implant geometry, but also on the material stiffness of the plate and screws and their load transfer through the bone-implant interfaces. Material properties used in the finite element models were derived from previously published studies (Liu et al., 2019; Yang et al., 2023; Luo et al., 2025; Weng et al., 2025) (Table 2). Contact interactions between cancellous bone and cortical bone, bone and cartilage, screws and plates, plates and bone, and screws and bone were all set as bonded.

**TABLE 2:** Material parameters of different materials in the finite element model.

Structure	Young’s modulus (MPa)	Poisson’s ratio
Sacral cortical bone	17,000	0.3
Sacral cancellous bone	132	0.2
Iliac cortical bone	6,140	0.3
Iliac cancellous bone	1,400	0.3
Femur	18,200	0.38
Sacroiliac cartilage	54	0.4
Pubic symphysis	5	0.45
Acetabular cartilage	12	0.42
Plates and screws	110,000	0.3

**FIGURE 2:**
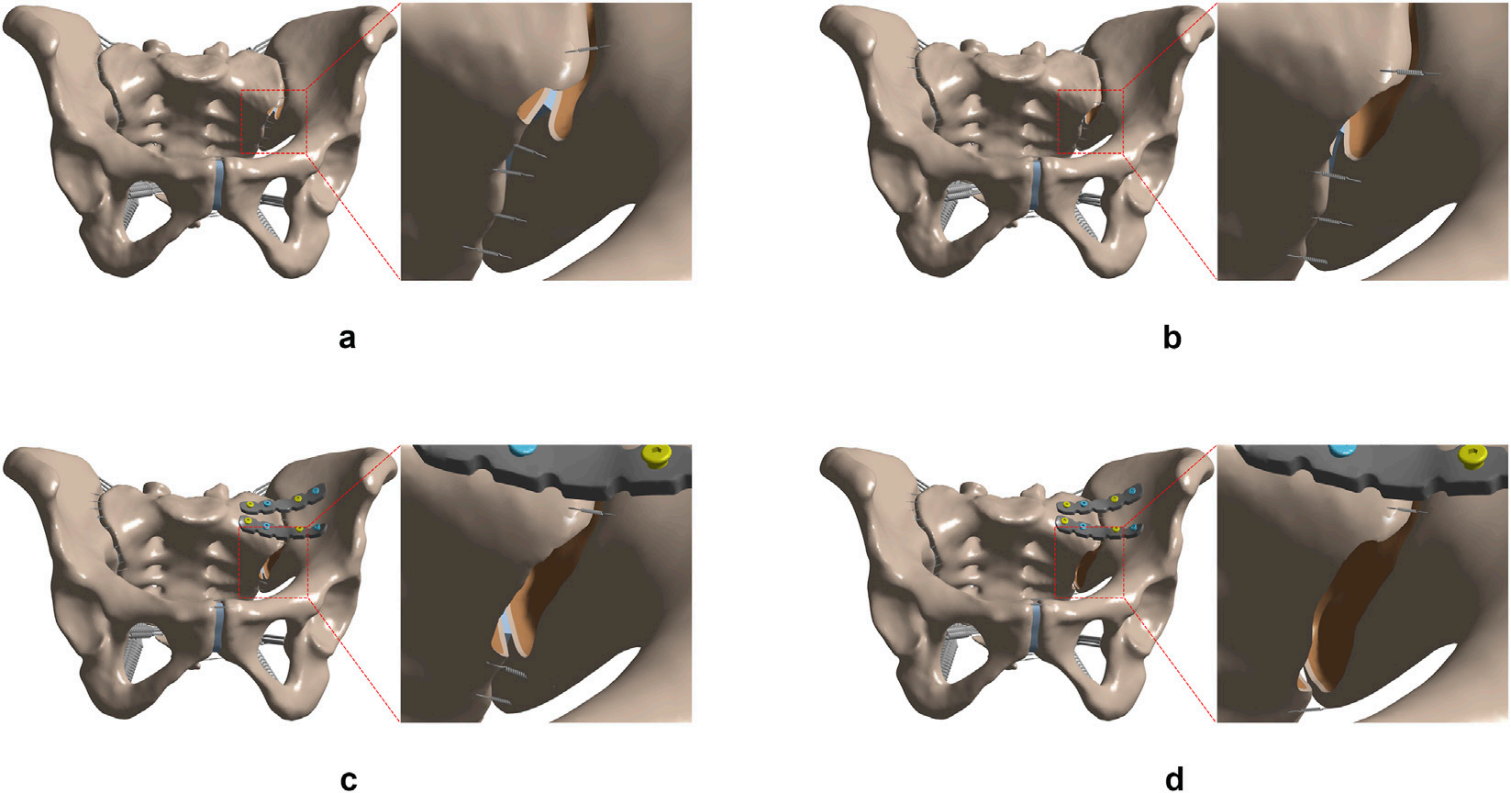
Postoperative finite element models under different degrees of destruction. **(a)** Lesion debridement-alone model with 25% destruction. **(b)** Lesion debridement-alone model with 50% destruction. **(c)** Lesion debridement combined with internal fixation model with 75% destruction. **(d)** Lesion debridement combined with internal fixation model with 100% destruction.

Figure 2 illustrates four representative postoperative finite element models. Figures 2a,b show the lesion debridement-alone models with 25% and 50% degrees of destruction, respectively, whereas Figures 2c,d present the lesion debridement combined with internal fixation models with 75% and 100% degrees of destruction, respectively. The remaining postoperative models with different surgical strategies at the same degrees of destruction were independently constructed using the same modeling workflow (Supplementary Figure S1).

#### 2.1.4 Finite element simulation analysis

Finite element analysis was performed using ANSYS 2022 (ANSYS Inc., Canonsburg, PA, USA). To simulate the mechanical environment of the pelvis in the standing position, the inferior surfaces of the distal ends of both femora were fixed, constraining translational and rotational degrees of freedom in all three directions. Based on human load-bearing capacity and previous biomechanical studies (Ramezani et al., 2019; Zhao et al., 2024), a 500 N axial compressive load was applied to the superior endplate of the sacrum at S1 to simulate physiological body weight transmission to the pelvis. Under these boundary conditions and loading scenarios, calculations were conducted for the intact pelvic model and the different postoperative models, and displacement and stress distribution characteristics of the overall pelvis and the sacroiliac joint region were systematically analyzed. All simulations were conducted as static analyses under a single quasi-static 500 N standing load to evaluate initial postoperative pelvic stability. Cyclic loading, gait simulation, and fatigue-related failure criteria were beyond the scope of this study.

#### 2.1.5 Observation parameters

This study primarily analyzed the overall and local displacement and stress of the pelvis under different surgical strategies, as well as the resistance of the sacroiliac joint to joint separation force, horizontal shear force, and vertical shear force. The outcome measures included the following categories.

##### 2.1.5.1 Global pelvic displacement and stress distribution

The global maximum displacement of the pelvis was compared among different models and degrees of destruction to evaluate the global mechanical response of the pelvis under physiological loading conditions. An increase in global displacement indicates a reduced ability of the pelvic structure to resist external loads, reflecting decreased pelvic stability.

##### 2.1.5.2 Relative displacement of the affected sacroiliac joint surface

According to the CT scanning orientation and the standard anatomical posture of the subject, the global Cartesian coordinate system in ANSYS was adopted as the reference coordinate system. The X-axis was oriented anteriorly toward the sacrum, the Y-axis toward the left side of the sacrum, and the Z-axis superiorly toward the sacrum. Accordingly, the plane defined by the Y- and Z-axes corresponded to the sagittal plane of the subject, and the X-axis was perpendicular to the sagittal plane.

**FIGURE 3:**
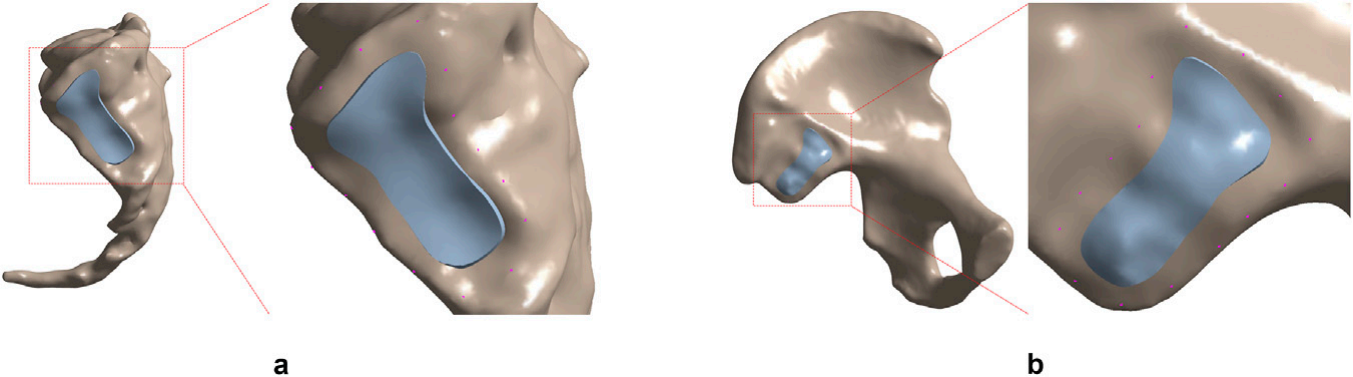
Distribution of corresponding measurement points around the auricular surface of the sacroiliac joint. **(a)** Distribution of measurement points on the sacral side. **(b)** Distribution of measurement points on the iliac side.

In the affected sacroiliac joint region, 15 corresponding observation points were selected on the sacral-side articular surface and the corresponding iliac-side articular surface, respectively. These points were evenly distributed along the sacroiliac joint surface to represent the mechanical behavior of the joint region (Figure 3). Paired observation points were selected to be approximately aligned in the horizontal direction, such that points with similar Y- and Z-coordinate values were grouped as a pair. This arrangement facilitated the calculation of relative displacement between the two sides of the sacroiliac joint and allowed evaluation of local stability of the affected joint. The three-dimensional displacements of the observation points were recorded as the coordinates of the sacral-side points (X_1_, Y_1_, Z_1_) and the corresponding iliac-side points (X_2_, Y_2_, Z_2_).

By calculating the relative displacement of each pair of corresponding observation points along the X-, Y-, and Z-directions, micromotion of the affected sacroiliac joint was evaluated. The calculation formula is as follows:

$$\Delta X=\left|X1-X_{2}\right|$$

$$\Delta Y=\left|Y_{1}-Y_{2}\right|$$

$$\Delta Z=\left|Z_{1}-Z_{2}\right|$$

The total relative displacement was calculated using the Euclidean norm and denoted as T, the calculation formula is as follows:

$$T=\sqrt{\left(\Delta X^{2}+\Delta Y^{2}+\Delta Z^{2}\right)}$$

This parameter directly reflects joint stability by measuring the relative displacement of 15 pairs of points between the sacral and iliac articular surfaces under physiological double-leg standing loading conditions, with smaller values indicating greater stability.

- Relative displacement in the X-direction primarily evaluates the ability of the sacroiliac joint to resist horizontal separation.
- Relative displacement in the Y-direction evaluates the ability of the sacroiliac joint to resist horizontal shear forces.
- Relative displacement in the Z-direction evaluates the ability of the sacroiliac joint to resist vertical shear force.
- The total relative displacement is used to comprehensively reflect the overall micromotion level of the affected sacroiliac joint under loading conditions.

##### 2.1.5.3 Displacement and stress distribution of the affected sacrum and ilium

To further evaluate the mechanical behavior of the pelvis under different surgical strategies and degrees of destruction, quantitative analyses of displacement and stress distribution were performed for the sacrum and the affected ilium. Under physiological loading conditions, the maximum displacement of the affected sacrum and ilium in each model was recorded to characterize the overall mechanical response of the local bony structures to external loads. An increase in maximum displacement reflects a reduction in local structural stiffness, indicating a corresponding decrease in stability. In addition, the maximum von Mises stress of the affected sacrum and ilium was extracted to assess stress distribution characteristics of the bone tissue under different reconstruction strategies and to evaluate the potential risk of stress concentration.

### 2.2 Clinical study

#### 2.2.1 Object of study

A retrospective analysis was conducted on patients who underwent lesion debridement for sacroiliac joint tuberculosis at our institution between January 2010 and December 2025. All patients underwent preoperative X-ray, CT, and MRI examinations, and the extent of sacroiliac joint involvement was comprehensively evaluated. Based on the proportion of articular surface involvement, the degree of joint destruction was classified as mild (<1/3), moderate (1/3–2/3), or severe (>2/3). Only patients with imaging-confirmed mild destruction were included in this study. According to whether internal fixation was performed intraoperatively, patients were divided into two groups: Group A (debridement-alone group) and Group B (debridement combined with internal fixation group), with 10 patients in each group. Inclusion criteria: (1) confirmed diagnosis of unilateral sacroiliac joint tuberculosis; (2) imaging showing joint destruction <1/3; (3) treatment with lesion debridement; and (4) complete clinical and follow-up data. Exclusion criteria: (1) severe pelvic instability or cases requiring complex reconstruction; (2) concomitant multifocal osteoarticular tuberculosis or disseminated tuberculosis; (3) prior sacroiliac joint surgery; and (4) loss to follow-up or incomplete key data.

#### 2.2.2 Preoperative preparation

All patients received standardized anti-tuberculosis therapy (HRZE regimen) for 2–4 weeks preoperatively to control disease activity. Routine preoperative evaluations included complete blood count, inflammatory markers (ESR and CRP), liver and renal function, and coagulation profiles. Pelvic X-ray, CT, and MRI were performed to assess lesion extent and the degree of bone destruction. For patients with abscess formation or marked inflammatory response, individualized preoperative management (e.g., drainage or intensified anti-infective therapy) was implemented. In addition, perioperative management plans were tailored according to the patients’ general condition, and blood was prepared preoperatively as needed.

#### 2.2.3 Operative procedures

All patients underwent posterior sacroiliac joint lesion debridement under general anesthesia. Through a posterior approach, the sacroiliac joint region was exposed, and tuberculous lesions, necrotic bone, and inflammatory granulation tissue were thoroughly debrided, followed by irrigation and bone grafting reconstruction. In Group A, only debridement and bone grafting were performed. In Group B, internal fixation was additionally applied to reconstruct sacroiliac joint stability following debridement. Care was taken to protect surrounding neurovascular structures throughout the procedure.

#### 2.2.4 Postoperative management

Postoperatively, all patients continued standardized anti-tuberculosis therapy for 12–18 months. Routine management included anti-infective treatment, analgesia, and nutritional support, along with monitoring of the surgical incision and drainage output. Drainage tubes were removed as appropriate based on clinical assessment. Early postoperative bed rest was recommended, followed by gradual mobilization under brace protection. Partial weight-bearing was initiated progressively according to recovery status, with transition to full weight-bearing after radiographic confirmation of bone graft fusion.

#### 2.2.5 Postoperative follow-up and observation parameters

All patients were followed up regularly, and clinical data were recorded at preoperative and postoperative time points. Outcome measures included: (1) operative time, intraoperative blood loss, and changes in inflammatory markers (ESR and CRP); (2) pain assessment using the VAS preoperatively and at final follow-up; (3) functional evaluation using the Majeed scoring system; (4) time to partial weight-bearing and bone graft healing; and (5) radiographic assessment of sacroiliac joint stability and bone fusion using X-ray or CT.

#### 2.2.6 Statistical method

All statistical analyses were performed using R software (version 4.5.2). Continuous variables were tested for normality (Shapiro-Wilk test) and homogeneity of variance (Levene’s test). Data with normal distribution and homogeneity of variance were expressed as mean ± standard deviation ( $\bar{x}\pm s$ ). Comparisons between two groups were conducted using independent-samples t-tests, while comparisons among multiple groups were performed using one-way analysis of variance (ANOVA), followed by Bonferroni *post hoc* multiple comparisons. Non-normally distributed data were expressed as median (interquartile range) and analyzed using the Mann-Whitney U test or Kruskal–Wallis test, with Dunn’s test for pairwise comparisons. A *P* value <0.05 was considered statistically significant.

## 3 Results

### 3.1 Finite element analysis

#### 3.1.1 Model validation

**FIGURE 4:**
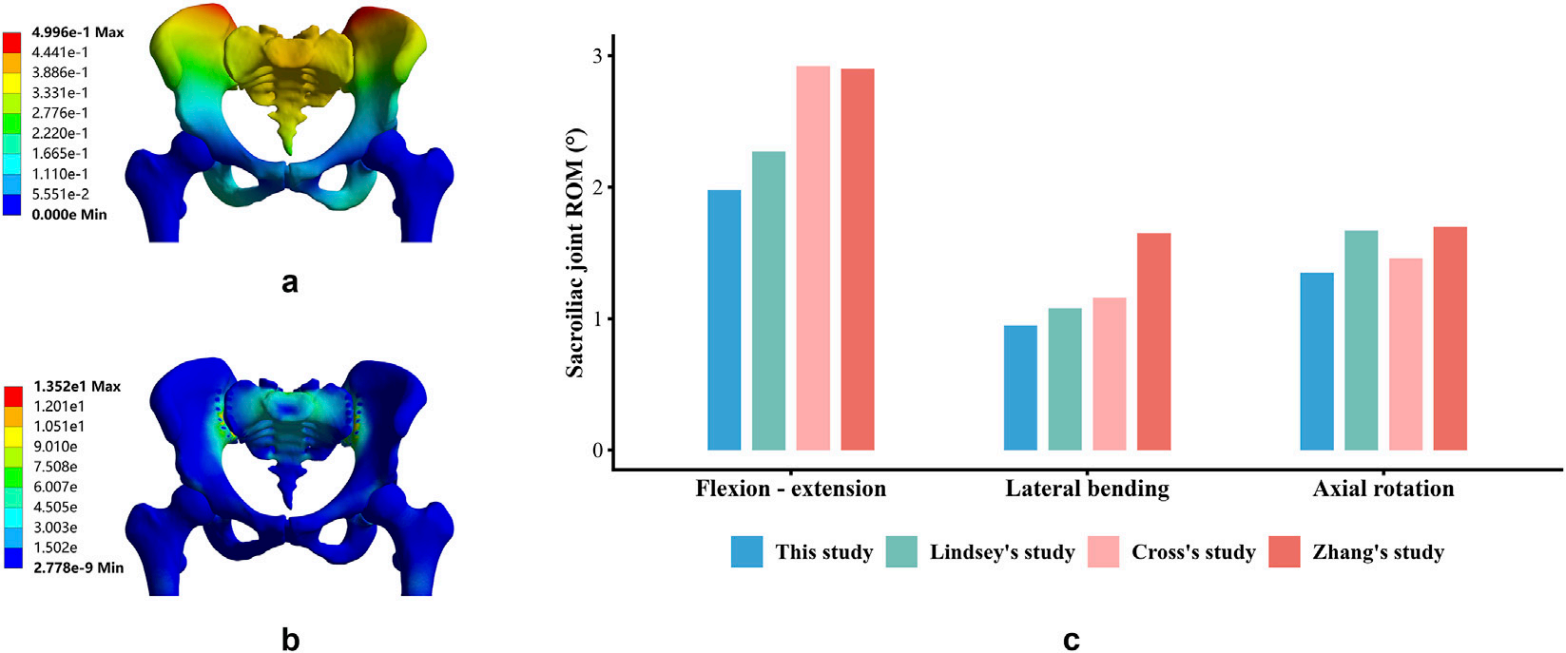
**(a)** Contour plot of maximum displacement in the intact pelvic model. **(b)** Contour plot of maximum stress in the intact pelvic model. **(c)** Comparison of the sacroiliac joint range of motion (ROM) in the intact finite element model established in this study with previously reported data.

The stress distribution and displacement characteristics of the intact pelvic finite element model under physiological loading conditions are shown in Figures 4a,b. The overall stress and displacement distributions exhibited an approximately symmetric pattern. The maximum total displacement was mainly concentrated in the bilateral iliac crest regions, whereas the maximum equivalent stress was located in the iliac arch region. The maximum displacement, maximum equivalent stress, and range of motion (ROM) of the sacroiliac joint obtained from the intact pelvic model were compared with those reported in previous *in vitro* biomechanical experiments and finite element studies (Hu et al., 2017; Cross et al., 2018; Zhang J. et al., 2024; Lindsey et al., 2014) (Figure 4c). The results demonstrated that all mechanical parameters were within comparable ranges. Although certain numerical differences were observed, which may be attributed to individual variability, model parameter settings, and numerical processing methods, the overall trends were highly consistent with those reported in the literature, supporting the rationality and validity of the finite element model established in this study.

#### 3.1.2 Analysis of global maximum pelvic displacement under different degrees of destruction

**FIGURE 5:**
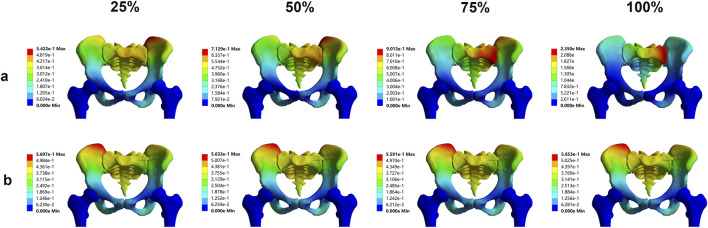
Contour plots of global pelvic displacement under different degrees of sacroiliac joint destruction. **(a)** Lesion debridement-alone models. **(b)** Lesion debridement combined with internal fixation models.

Under axial loading conditions, the global maximum displacement of the pelvis in the lesion debridement-alone models exhibited a progressive increasing trend as the degree of sacroiliac joint destruction increased from 25% to 100% (Figure 5a). Compared with the intact pelvic model, the maximum displacement increased by approximately 8.5%, 42.7%, 80.4%, and 370.3% in the models with 25%, 50%, 75%, and 100% destruction, respectively. In cases of 25% destruction, regions of high displacement were mainly confined to the superior portion of the affected sacrum and the sacral ala. With increasing destruction severity, these regions gradually extended to both sides of the sacrum and toward the sacral midline. This finding indicates that sacroiliac joint structural damage not only increases displacement magnitude but also markedly expands the extent of global pelvic deformation. After internal fixation, the global maximum displacement was substantially reduced across all degrees of destruction. Compared with the intact pelvis, the increase in maximum displacement in the fixation models was limited to approximately 12%–13%. In addition, regions of high displacement were markedly reduced in extent and were mainly confined to the iliac ala on the unaffected side (Figure 5b).

#### 3.1.3 Relative displacement of the articular surface under different degrees of destruction

Based on the 15 pairs of corresponding points on the sacroiliac joint surface, relative displacement of the articular surface was calculated under different degrees of destruction. Comparisons were performed among the intact model, the lesion debridement-alone models, and the internal fixation models with respect to relative displacement in the X-, Y-, and Z-directions, as well as the total relative displacement.

##### 3.1.3.1 Relative articular surface displacement under 25% destruction

Under the condition of 25% sacroiliac joint destruction, relative articular surface displacement in the X-, Y-, and Z-directions, as well as the total relative displacement (T), was generally small across all three models (Supplementary Table S1). The displacement magnitudes of the intact model and the lesion debridement-alone model were comparable, while the internal fixation model exhibited slightly lower relative displacement in all directions; however, the differences were limited. The results of the Kruskal–Wallis H test showed that no statistically significant differences were observed among the three models in any directional displacement or in the total relative displacement (*P* > 0.05). These findings indicate that at the stage of mild destruction (25%), overall sacroiliac joint stability is largely preserved, and different treatment strategies have no significant effect on relative articular surface displacement.

##### 3.1.3.2 Relative articular surface displacement under 50% destruction

When the degree of destruction increased to 50%, the lesion debridement-alone model exhibited further increases in relative displacement in the X-, Y-, and Z-directions, as well as in the total relative displacement (T), compared with the 25% destruction condition. The increases in the Z-direction and in the total relative displacement were the most pronounced, indicating that with progressive articular surface destruction, impairment of sacroiliac joint stability in the vertical direction and in overall stability became more evident. In contrast, the internal fixation model maintained relatively low relative displacement in all directions, with values overall close to those of the intact model. Statistical analysis demonstrated that differences among the three models in the X-, Y-, Z-directions and in the total relative displacement were statistically significant (*P* < 0.05) (Supplementary Table S2).

##### 3.1.3.3 Relative articular surface displacement under 75% destruction

Under the 75% destruction condition, relative articular surface displacement in the lesion debridement-alone model was further amplified, exhibiting a pattern of progressive instability. Compared with the 50% destruction condition, relative displacement in the Z-direction and the total relative displacement (T) continued to increase, indicating that as articular surface integrity was further compromised, relative motion of the sacroiliac joint in the primary load-bearing direction became markedly aggravated. Meanwhile, relative displacement in the internal fixation model remained significantly lower than that in the lesion debridement-alone model in all directions, with values maintained within a range close to those of the intact model. Statistical analysis demonstrated that differences among the three models in the X-, Y-, Z-directions and in the total relative displacement were statistically significant (*P* < 0.001) (Supplementary Table S3).

##### 3.1.3.4 Relative articular surface displacement under 100% destruction

Under the condition of complete (100%) destruction, the lesion debridement-alone model exhibited the highest level of relative articular surface displacement. Relative displacement in the Z-direction and the total relative displacement (T) increased markedly, indicating that the sacroiliac joint had entered a state of pronounced instability. At this stage, relative displacement in all directions was substantially greater than that observed in models with lower degrees of destruction, reflecting the severe impact of complete articular surface destruction on overall joint stability. In contrast, the internal fixation model maintained relatively low relative displacement in all directions, with values significantly lower than those of the lesion debridement-alone model. Statistical analysis demonstrated that differences among the three models in the X-, Y-, Z-directions and in the total relative displacement were highly statistically significant (*P* < 0.001) (Supplementary Table S4).

#### 3.1.4 Local displacement characteristics of the affected sacroiliac joint under different degrees of destruction

##### 3.1.4.1 Displacement characteristics of the affected ilium under different degrees of destruction

**FIGURE 6:**
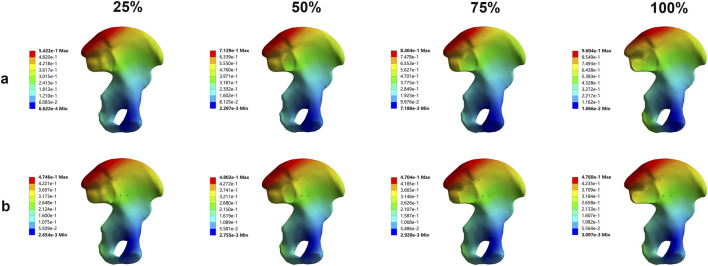
Contour plots of maximum displacement of the affected ilium under different degrees of destruction (mm). **(a)** Lesion debridement-alone models. **(b)** Internal fixation models.

Compared with the intact ilium model (Supplementary Figure S2a), the maximum displacement of the ilium under lesion debridement-alone conditions increased progressively with increasing degrees of destruction, showing increases of approximately 8.5%, 42.7%, 68.2%, and 92.2% under 25%, 50%, 75%, and 100% destruction, respectively. These findings indicate that structural damage to the sacroiliac joint can markedly impair the mechanical stability of the ilium (Figure 6a). After internal fixation at the same degrees of destruction, the maximum displacement of the ilium was consistently lower than that of the corresponding lesion debridement-alone models (Figure 6b). Moreover, as the degree of destruction increased, the displacement-restraining effect of internal fixation became progressively more pronounced, suggesting that internal fixation effectively limits abnormal displacement of the affected ilium and provides a more substantial stabilizing effect under moderate to severe destruction conditions (≥50% destruction).

##### 3.1.4.2 Displacement characteristics of the affected sacrum under different degrees of destruction

**FIGURE 7:**
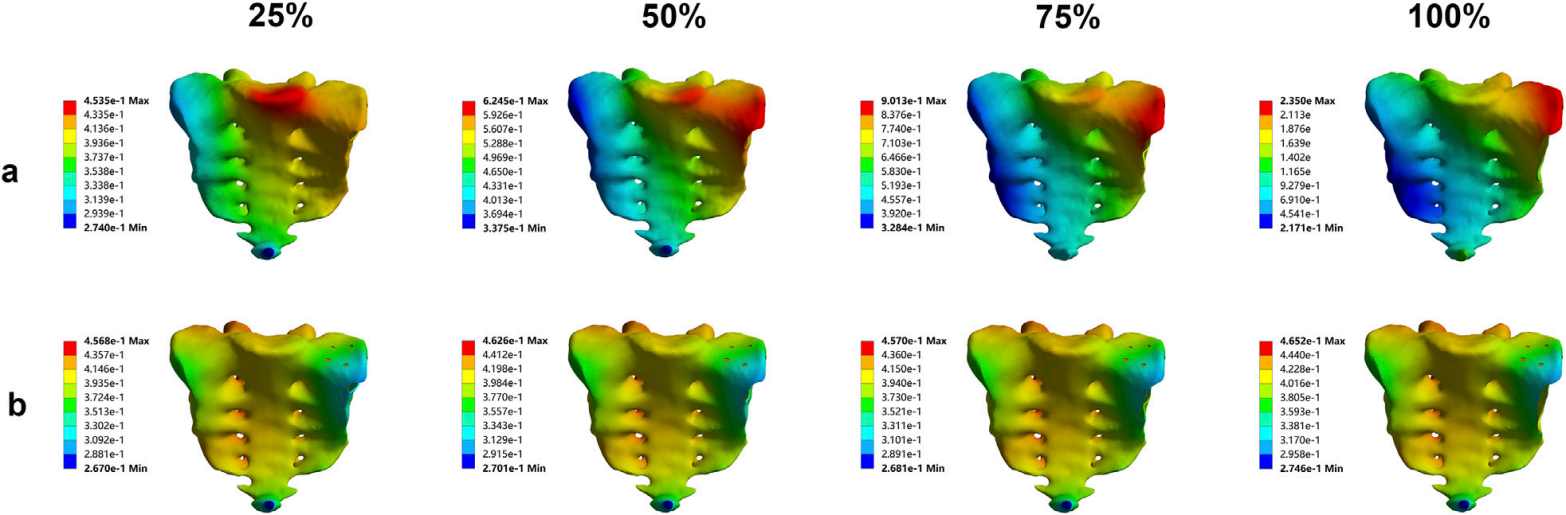
Contour plots of maximum displacement of the affected sacrum under different degrees of destruction (mm). **(a)** Lesion debridement-alone models. **(b)** Internal fixation models.

Compared with the intact sacrum model (Supplementary Figure S2b), the maximum displacement of the sacrum under lesion debridement-alone conditions increased markedly with increasing degrees of sacroiliac joint destruction. Under 25%, 50%, 75%, and 100% destruction, the maximum displacement increased by approximately 8.4%, 49.2%, 115.4%, and 461.5%, respectively, indicating that structural damage to the sacroiliac joint can substantially impair the mechanical stability of the sacrum (Figure 7). After internal fixation at the same degrees of destruction, the maximum displacement of the sacrum was significantly reduced compared with the corresponding lesion debridement-alone models. Except for the mild destruction condition (25%), displacement was reduced by approximately 25.9%, 49.3%, and 80.2% under 50%, 75%, and 100% destruction, respectively. Moreover, as the degree of destruction increased, the inhibitory effect of internal fixation on abnormal sacral displacement became progressively more pronounced, indicating that internal fixation can markedly improve local mechanical stability of the sacrum under moderate to severe destruction conditions (≥50% destruction).

#### 3.1.5 Local stress distribution of the affected sacroiliac joint under different degrees of destruction

##### 3.1.5.1 Maximum sacral stress under different degrees of destruction

**FIGURE 8:**
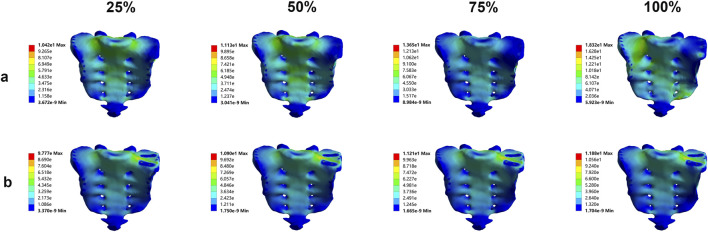
Contour plots of equivalent stress distribution of the affected sacrum under different degrees of sacroiliac joint destruction (MPa). **(a)** Lesion debridement-alone models. **(b)** Internal fixation models.

Under axial loading conditions, the maximum equivalent stress of the sacrum in the lesion debridement-alone models showed a progressive increase with increasing degrees of sacroiliac joint destruction. Using the intact model as a reference (Supplementary Figure S2c), the maximum stress under 25%, 50%, 75%, and 100% destruction was approximately 1.15, 1.22, 1.50, and 2.01 times that of the intact model, respectively, with the most pronounced stress amplification observed at the 75% and complete destruction stages (Figure 8a). In the internal fixation models, the maximum equivalent stress of the sacrum at all degrees of destruction was markedly lower than that in the corresponding lesion debridement-alone models and remained overall close to the level of the intact model. In addition, the stress distribution exhibited an approximately symmetric pattern (Figure 8b).

##### 3.1.5.2 Maximum iliac stress under different degrees of destruction

**FIGURE 9:**
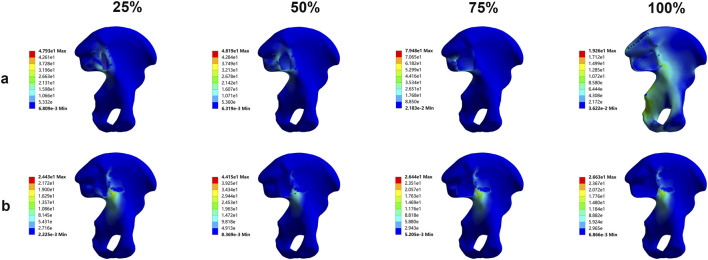
Contour plots of equivalent stress distribution of the affected ilium under different degrees of sacroiliac joint destruction (MPa). **(a)** Lesion debridement-alone models. **(b)** Internal fixation models.

In the lesion debridement-alone models, the maximum equivalent stress of the affected ilium showed a distinct variation pattern with increasing degrees of sacroiliac joint destruction (Figure 9a). Compared with the intact model (Supplementary Figure S2d), the maximum stress increased to approximately 3.5 times that of the intact model under 25% and 50% destruction and further increased to approximately 5.9 times under 75% destruction. In contrast, under the condition of complete (100%) destruction, the maximum stress decreased and was only about 1.4 times that of the intact model, suggesting an alteration in the overall load transfer pathway. In the internal fixation models, the maximum stress of the affected ilium at each degree of destruction was generally lower than that of the corresponding lesion debridement-alone models and did not show a continuous increase with increasing destruction severity (Figure 9b).

### 3.2 Clinical research

#### 3.2.1 General condition

A total of 20 patients were included, all of whom underwent surgical treatment and completed follow-up. Baseline characteristics were comparable between the two groups (*P* > 0.05) (Table 3). Group A consisted of 10 patients (5 males and 5 females; age range, 21–75 years), while Group B included 10 patients (4 males and 6 females; age range, 19–70 years; mean age, 43.2 years). The disease duration in Group A was 7.10 ± 3.48 months, with a median operative time of 123.00 (113.50, 129.50) minutes and an intraoperative blood loss of 251.00 ± 59.90 mL. In Group B, the disease duration was 9.80 ± 2.82 months, with a median operative time of 160.50 (145.50, 185.75) minutes and an intraoperative blood loss of 337.00 ± 115.38 mL. Operative time was significantly longer in Group B than in Group A (*P* < 0.05), whereas no significant differences were observed in disease duration or intraoperative blood loss between the two groups (*P* > 0.05).

**TABLE 3:** Baseline characteristics of patients in Groups A and B.

Items	Group A	Group B	Test value (*t/u/χ* ^*2*^)	*P* value
Age (years)	40.80 ± 20.01	43.20 ± 20.57	−0.26	0.85
Sex (male/female)	5/5	4/6	0.20	0.65
Disease duration (months)	7.10 ± 3.48	9.80 ± 2.82	−1.91	0.07
Operative time (min)	123.00 (113.50, 129.50)	160.50 (145.50, 185.75)	5.00	<0.001
Intraoperative blood loss (mL)	251.00 ± 59.90	337.00 ± 115.38	−2.09	0.051

#### 3.2.2 Comparison of ESR and CRP levels

Postoperative inflammatory markers (ESR and CRP) decreased progressively in both groups, indicating effective infection control following anti-tuberculosis therapy and surgical debridement. No significant differences were observed between the two groups at any time point (*P* > 0.05) (Table 4).

**TABLE 4:** Comparison of ESR and CRP levels between Groups A and B.

​	ESR		CRP	
Items	Preoperative	3 months postoperatively	Preoperative	3 months postoperatively
Group A	57.90 ± 24.30	18.10 ± 8.41	32.99 ± 21.06	11.21 ± 3.73
Group B	51.73 ± 39.33	14.60 ± 7.03	38.79 ± 32.87	10.82 ± 8.42
*t*	0.42	1.01	−0.47	0.13
*P*	0.68	0.33	0.64	0.90

#### 3.2.3 Pain relief

VAS scores decreased gradually over time in both groups, suggesting that surgical treatment effectively alleviated pain. There were no significant differences in VAS scores between the two groups at any preoperative or postoperative time point (*P* > 0.05) (Table 5).

**TABLE 5:** Comparison of VAS scores between Groups A and B.

Items	Preoperative	Immediate postoperative	3 months postoperatively	6 months postoperatively
Group A	6.50 (6.00, 7.00)	5.50 (5.00, 6.00) ^*^	3.00 (3.00, 3.75) ^*#^	1.00 (0.25, 1.75) ^*#†^
Group B	6.00 (6.00, 7.00)	5.50 (5.00, 6.00) ^*^	3.00 (2.25, 3.00) ^*#^	0.50 (0.00, 1.00) ^*#†^
*u*	54.50	52.50	58.50	64.00
*P*	0.74	0.87	0.51	0.27

*P < 0.05 vs. preoperative; #*P* < 0.05 vs. immediate postoperative; †*P* < 0.05 vs. 3 months postoperatively.

#### 3.2.4 Functional recovery

Majeed scores improved progressively during follow-up in both groups, indicating effective restoration of pelvic function. No significant differences were observed between the two groups at any time point (*P* > 0.05) (Table 6).

**TABLE 6:** Comparison of Majeed scores between Groups A and B.

Items	Preoperative	3 months postoperatively	6 months postoperatively
Group A	53.60 ± 8.29	72.40 ± 4.30^*^	84.40 ± 3.31^*#^
Group B	52.90 ± 9.00	70.30 ± 5.29^*^	85.00 ± 4.32^*#^
*t*	0.18	0.97	0.35
*P*	0.86	0.34	0.73

*P < 0.05 vs. preoperative; #*P* < 0.05 vs. 3 months postoperatively.

#### 3.2.5 Comparison of time to partial weight-bearing and bone graft fusion

During postoperative rehabilitation, patients in both groups gradually regained weight-bearing function and achieved bone graft fusion. Compared with the debridement-alone group (Group A), the debridement combined with internal fixation group (Group B) showed no significant advantages in time to partial weight-bearing or bone graft fusion (*P* > 0.05) (Table 7). These findings suggest that, in patients with mild sacroiliac joint destruction, adjunctive internal fixation does not significantly shorten the time to postoperative weight-bearing recovery or bone graft fusion.

**TABLE 7:** Comparison of time to partial weight-bearing and bone graft fusion between Groups A and B.

Items	Time to partial weight-bearing (days)	Time to bone graft fusion (months)
Group A	10.00 ± 1.76	7.00 ± 1.25
Group B	11.40 ± 2.32	7.30 ± 1.16
*t*	−1.52	−0.56
*P*	0.15	0.58

#### 3.2.6 Complications

No severe perioperative complications were observed during follow-up in either group. In Group A, no recurrence of infection, poor wound healing, or pelvic instability was noted. In Group B, no fixation-related complications, such as implant breakage, loosening, or displacement, were observed, and implant positioning remained satisfactory. Mild postoperative complications occurred in three patients, including transient incision pain, mild anemia, and low-grade fever, all of which resolved with symptomatic treatment and did not affect final outcomes. To further illustrate treatment outcomes, two representative cases are presented: one patient with mild destruction treated with debridement and bone grafting (Supplementary Figure S3), and one patient with mild destruction treated with debridement combined with internal fixation (Supplementary Figure S4).

## 4 Discussion

Sacroiliac joint tuberculosis is a chronic osteodestructive disease involving the articular cartilage and adjacent bone, and its progression is often accompanied by impairment of joint structural integrity (Tian et al., 2022b). For patients with extensive joint involvement or poor response to conservative treatment, surgical intervention represents an important therapeutic option. However, residual bone defects following lesion debridement may alter load transfer pathways of the sacroiliac joint, thereby affecting the overall biomechanical environment of the pelvis (Zheng et al., 2014). At present, the patterns of postoperative pelvic stability under different degrees of destruction, particularly the threshold at which additional internal fixation becomes necessary, remain unclear due to a lack of definitive biomechanical evidence. In this study, finite element analysis combined with retrospective clinical data was used to evaluate postoperative biomechanical changes at varying degrees of destruction and to assess the clinical value of internal fixation in patients with mild destruction. The results demonstrated that the degree of sacroiliac joint destruction is closely associated with postoperative pelvic stability, with stability progressively declining as destruction increases. In contrast, among patients with mild destruction, no significant differences were observed between debridement alone and debridement combined with internal fixation in terms of pain relief, functional recovery, or postoperative rehabilitation, suggesting a stage-dependent role of internal fixation.

Finite element analysis demonstrated a clear stage-dependent relationship between the degree of sacroiliac joint destruction and pelvic stability. With increasing destruction, overall pelvic displacement, relative displacement across the joint surface, and local displacement and stress in the sacrum and ilium progressively increased, indicating a reduced capacity of the pelvic ring to resist axial loading. Previous studies (Guo et al., 2022; Tian et al., 2022b; Zhao et al., 2025) have shown that pelvic stability mainly depends on the congruency of the sacroiliac joint, osseous support, and the coordinated function of surrounding ligamentous structures, with only limited micromotion under normal conditions. In the present study, under mild destruction, the residual bony structures were sufficient to maintain primary load transfer pathways, resulting in minimal changes in biomechanical parameters and preservation of overall stability. As destruction progressed, loss of joint congruency and reduced osseous support altered load transmission within the pelvic ring, leading to significant increases in overall displacement and abnormal micromotion, particularly in vertical displacement and total relative displacement, reflecting a marked decline in the ability to resist axial and shear forces. These findings are consistent with previous studies reporting reduced stiffness and compromised stability following sacroiliac joint structural damage (Falowski and Valdevit, 2022; Zhao et al., 2025; Zhang et al., 2025). Meanwhile, with increasing destruction, local stress in the sacrum and ilium progressively increased and exhibited stress concentration, indicating a more uneven load distribution and greater mechanical demand on local structures, thereby reducing stability and safety margins (Lee et al., 2017; Enix and Mayer, 2019). On this basis, the biomechanical role of internal fixation also exhibited a stage-dependent pattern. Under mild destruction, internal fixation provided limited improvement in pelvic displacement, relative joint displacement, and stress distribution, with no significant difference compared with the debridement-alone model. In contrast, under moderate-to-severe destruction, internal fixation significantly reduced overall displacement, restricted abnormal micromotion, and alleviated stress concentration, resulting in a more uniform mechanical environment. Mechanically, the fixation plate functions as a bridging element across the debrided sacroiliac region. By providing axial stiffness and flexural rigidity, the plate-screw construct participates in load sharing between the sacrum and ilium, reduces local bending and shear deformation, and limits abnormal micromotion at the affected sacroiliac joint. Therefore, the stabilizing effect of internal fixation observed in the finite element analysis can be explained not only by implant geometry, but also by the mechanical stiffness of the plate-screw construct. These findings are consistent with previous studies, suggesting that internal fixation enhances pelvic ring stiffness and restores stability by participating in load sharing (Venayre et al., 2021; Gonçalves et al., 2023). The present findings further suggest that this effect becomes more pronounced when structural integrity is substantially compromised, whereas its biomechanical contribution remains limited when residual structures are sufficient to maintain stability.

In the clinical part of this study, we included patients with imaging-confirmed mild destruction (articular surface involvement <1/3) and compared the clinical outcomes between debridement alone and debridement combined with internal fixation. The results showed no significant differences between the two groups in terms of pain relief (VAS score), functional recovery (Majeed score), inflammatory markers (ESR and CRP), or postoperative rehabilitation parameters, indicating comparable clinical efficacy between the two surgical strategies in patients with mild sacroiliac joint destruction. Previous studies (Tian et al., 2022b) have suggested that the key to the treatment of sacroiliac joint tuberculosis lies in thorough lesion debridement and effective infection control. Debridement combined with bone grafting can restore joint stability and achieve favorable clinical outcomes in most cases. Some studies (Tian et al., 2022a) have also reported that, in the absence of significant instability, additional internal fixation may not substantially improve functional recovery or accelerate rehabilitation. The findings of the present study are consistent with these reports, demonstrating that satisfactory outcomes can be achieved with debridement alone in patients with mild bone destruction, while routine adjunctive internal fixation does not confer clear additional benefits. Therefore, in patients with mild sacroiliac joint tuberculosis, surgical decision-making may place greater emphasis on the adequacy of debridement and infection control, whereas the use of internal fixation should be carefully individualized based on the specific clinical condition.

Although finite element outputs and clinical findings represent different types of data, their comparison revealed a consistent trend under mild sacroiliac joint destruction. Finite element results indicate that pelvic ring stability progressively declines with increasing structural destruction of the sacroiliac joint. Correspondingly, clinical outcomes show that, in patients with mild destruction, no significant differences are observed between surgical strategies in terms of pain relief, functional recovery, or postoperative rehabilitation. Although the finite element model employed idealized graded proportions of destruction, whereas clinical practice relies on semi-quantitative imaging-based assessment, both approaches consistently reflect the fundamental relationship between structural damage and stability changes. Based on these findings, the degree of sacroiliac joint destruction may represent a key factor influencing surgical decision-making. When structural damage is limited and overall stability can still be maintained, debridement alone appears sufficient to achieve satisfactory outcomes, and the routine use of adjunctive internal fixation should be approached with caution. As the degree of destruction increases, however, the stabilizing effect of internal fixation may become progressively more pronounced. Therefore, in clinical practice, the decision to apply internal fixation should not be based solely on the presence of bone destruction, but rather on a comprehensive assessment of the extent of damage and overall pelvic stability, thereby enabling more rational and individualized treatment strategies.

This study, combining finite element analysis with clinical data, investigated pelvic stability changes following lesion debridement at different degrees of sacroiliac joint destruction, as well as the biomechanical role of internal fixation. However, several limitations should be acknowledged. First, the finite element model was constructed based on a single healthy subject and therefore could not fully capture inter-individual variations in pelvic anatomy, bone density, and ligamentous properties. In addition, the postoperative debrided region was simplified as a mechanically non-load-bearing defect. Although this region was clinically filled with particulate bone grafts and may contain postoperative fluids such as blood, tissue fluid, and residual irrigation fluid, particulate grafts may not provide reliable immediate mechanical support before graft incorporation and fusion, and postoperative fluids cannot effectively sustain shear stress or transmit mechanical load. Therefore, these components were not explicitly modeled. Second, the classification of sacroiliac joint destruction was based on discrete intervals, which may be insufficient for precisely identifying the critical threshold at which pelvic stability significantly deteriorates. Third, the clinical component was a small-sample retrospective study including only patients with mild bone destruction; therefore, the clinical findings mainly support the biomechanical results under mild destruction, whereas the finite element predictions for moderate-to-severe destruction require further clinical validation.

Future studies should focus on improving the biomechanical realism of the finite element model and strengthening clinical validation. More patient-specific models incorporating variations in anatomy, bone quality, and lesion characteristics should be developed. In addition, dynamic or cyclic loading conditions should be introduced to better simulate physiological activities and evaluate long-term mechanical behavior. Finally, larger prospective clinical studies are needed to validate the biomechanical findings and further clarify the role of internal fixation in sacroiliac joint tuberculosis.

Taken together, the finite element analysis and clinical observations provide complementary evidence for understanding postoperative pelvic stability after lesion debridement for sacroiliac joint tuberculosis. The biomechanical model allowed quantitative assessment of displacement and stress changes under different degrees of joint destruction, whereas the clinical data provided preliminary outcome-based support for the model predictions under mild destruction. Although the finite element findings for moderate-to-severe destruction still require further clinical validation, the combined analytical and clinical approach offers a useful framework for exploring individualized surgical strategies in sacroiliac joint tuberculosis.

## 5 Conclusion

This study represents a first attempt to establish a biomechanical arithmetic modeling framework for sacroiliac joint tuberculosis after lesion debridement and to compare the model outputs with relevant clinical findings. By integrating finite element analysis with a retrospective clinical investigation, this study demonstrated that the degree of sacroiliac joint destruction is closely associated with postoperative pelvic stability. Under mild bone destruction, debridement alone is sufficient to maintain favorable biomechanical stability, with no clear additional clinical benefit observed from adjunctive internal fixation. In contrast, under moderate-to-severe destruction, combined internal fixation plays a more pronounced role in improving stress distribution, limiting abnormal displacement, and maintaining overall stability.

## Data Availability

The original contributions presented in the study are included in the article/Supplementary Material, further inquiries can be directed to the corresponding authors.
