# Supplementary material for: Biomechanical and clinical analysis of pelvic stability and the role of internal fixation after debridement for sacroiliac joint tuberculosis with different degrees of bone destruction
Source: Front Bioeng Biotechnol. 2026 Jul 21;14:1808745. doi: 10.3389/fbioe.2026.1808745 (PMC13433626; doi:10.3389/fbioe.2026.1808745)
Supplement: Supplementary file 1 [file datasheet1.pdf]

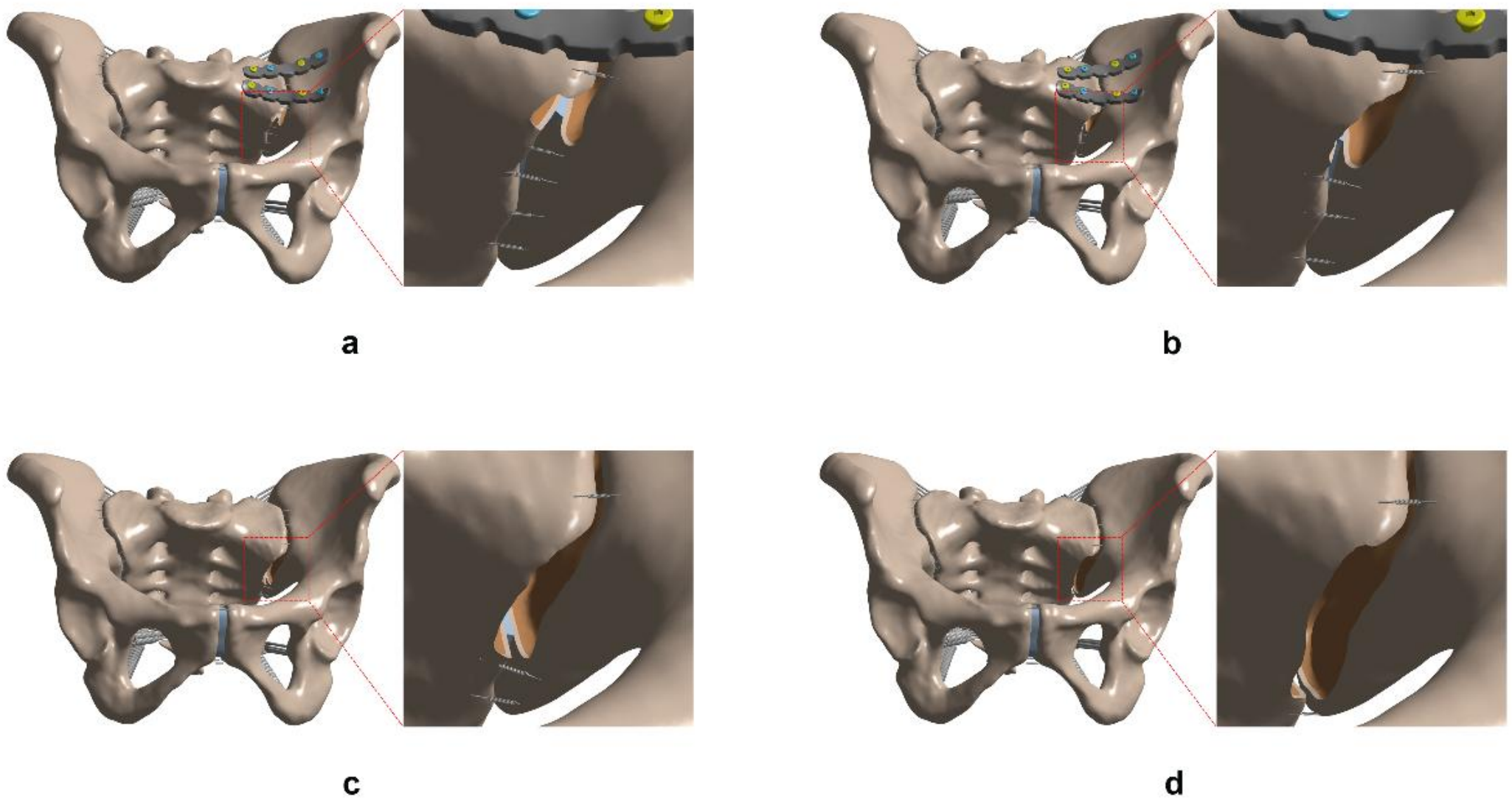

Supplementary Figure 1. Postoperative finite element models under different degrees of destruction. (a) Lesion debridement combined with plate internal fixation model with 25% destruction. (b) Lesion debridement combined with plate internal fixation model with 50% destruction. (c) Lesion debridement-only model with 75% destruction. (d) Lesion debridement-only model with 100% destruction.

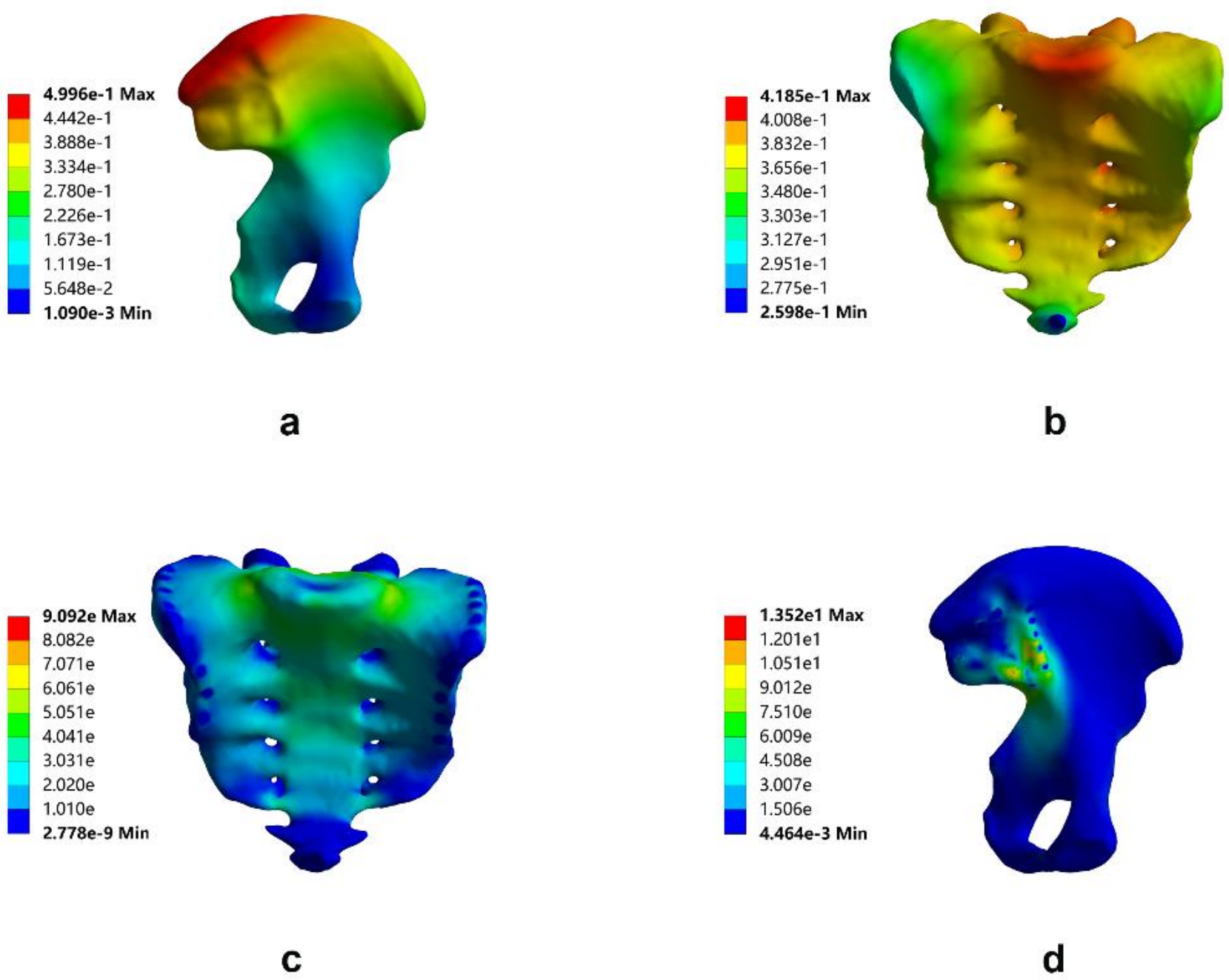

Supplementary Figure 2. Displacement and stress distribution of the intact ilium and sacrum model under physiological loading conditions. (a) Displacement distribution of the intact ilium. (b) Displacement distribution of the intact sacrum. (c) Von Mises stress distribution of the intact sacrum. (d) Von Mises stress distribution of the intact ilium.

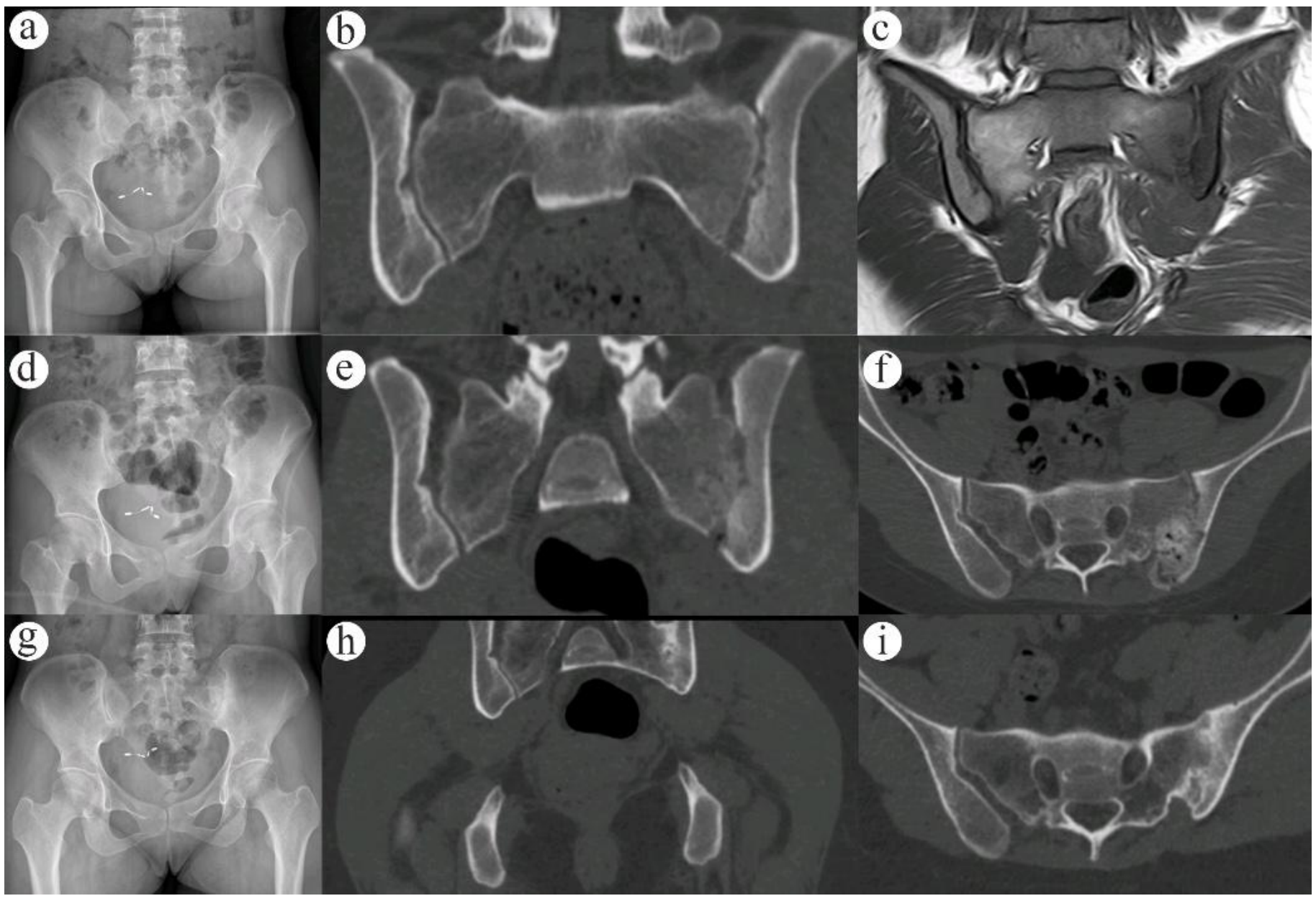

Supplementary Figure 3. Imaging of left sacroiliac joint tuberculosis treated with lesion debridement and bone grafting: a-c. Preoperative imaging, including pelvic X-rays, coronal CT, and MRI; d-f: Immediate postoperative imaging (X-ray, coronal CT, and axial CT); g-i. At 3-year follow-up, X-ray and CT images showed maintained pelvic stability, satisfactory bone fusion, and no evidence of recurrence.

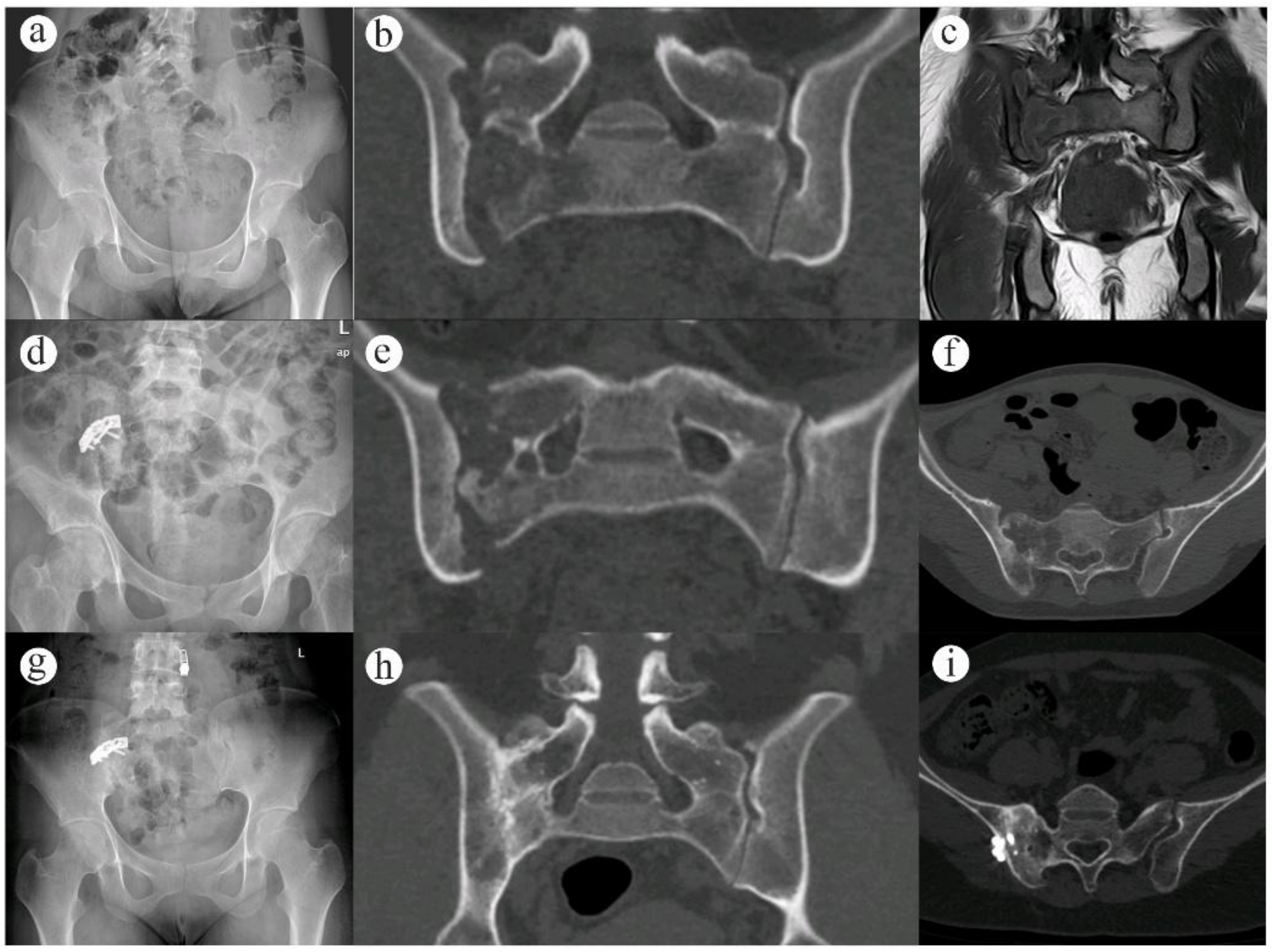

Supplementary Figure 4. Imaging of right sacroiliac joint tuberculosis treated with lesion debridement combined with internal fixation: a-c. Preoperative imaging, including pelvic X-rays, coronal CT, and MRI; d-f: Immediate postoperative imaging (X-ray, coronal CT, and axial CT); g-i. At 3-year follow-up, X-ray and CT images showed maintained pelvic stability, satisfactory bone fusion, and no evidence of recurrence.

Supplementary Table 1. Comparison of relative displacement of the sacroiliac joint surface among different models under 25% destruction (mm)

Model	X	Y	Z	T
Intact	0.01(0.01,0.03)	0.02(0.02,0.03)	0.02(0.02,0.06)	0.02(0.01,0.04)
Debridement-alone	0.02(0.01,0.03)	0.03(0.02,0.03)	0.03(0.02,0.05)	0.02(0.01,0.04)
Plate fixation	0.01(0.01,0.02)	0.02(0.02,0.02)	0.01(0.00,0.03)	0.01(0.00,0.02)
H value	4.67	4.81	5.74	5.68
P value	0.097	0.090	0.057	0.058

Supplementary Table 2. Comparison of relative displacement of the sacroiliac joint surface among different models under 50% destruction (mm)

Model	X	Y	Z	T
Intact	0.01(0.01,0.03)	0.02(0.02,0.03)	0.02(0.02,0.06)	0.02(0.01,0.04)
Debridement-alone	0.06(0.02,0.10) *	0.06(0.05,0.07) *	0.09(0.03,0.24)	0.06(0.03,0.12) *
Plate fixation	0.00(0.00,0.01) *#	0.01(0.00,0.02) #	0.01(0.00,0.02) #	0.01(0.00,0.02) #
H value	22.05	19.40	15.37	14.24
P value	<0.001	<0.001	<0.001	<0.001

Note: **P* < 0.05 vs. Intact; #*P* < 0.05 vs. Debridement-alone.

Supplementary Table 3. Comparison of relative displacement of the sacroiliac joint surface among different models under 75% destruction (mm)

Model	X	Y	Z	T
Intact	0.01(0.01,0.03)	0.02(0.02,0.03)	0.02(0.02,0.06)	0.02(0.01,0.04)
Debridement-alone	0.10(0.06,0.17) *	0.08(0.07,0.12) *	0.35(0.13,0.53) *	0.21(0.09,0.36) *
Plate fixation	0.00(0.00,0.01) #	0.01(0.01,0.03) #	0.01(0.00,0.02) #	0.01(0.01,0.03) #
H value	24.43	21.89	27.97	16.71
P value	<0.001	<0.001	<0.001	<0.001

Note: **P* < 0.05 vs. Intact; #*P* < 0.05 vs. Debridement-alone.

Supplementary Table 4. Comparison of relative displacement of the sacroiliac joint surface among different models under 100% destruction (mm)

Model	X	Y	Z	T
Intact	0.01(0.01,0.03)	0.02(0.02,0.03)	0.02(0.02,0.06)	0.02(0.01,0.04)
Debridement-alone	0.29(0.12,0.45) *	0.45(0.19,0.66) *	1.50(1.14,1.89) *	1.31(0.98,1.59) *
Plate fixation	0.00(0.00,0.01) #	0.02(0.01,0.04) #	0.01(0.01,0.02) #	0.02(0.01,0.05) #
H value	30.81	29.47	30.95	29.58
P value	<0.001	<0.001	<0.001	<0.001

Note: *$P < 0.05$ vs. Intact; #$P < 0.05$ vs. Debridement-alone.
